# Relationship between Prefecture-Level Yield of Not-for-Sale Fruits and Vegetables and Individual-Level Fruit and Vegetable Intake in Japan: A Cross-Sectional Study

**DOI:** 10.3390/nu13114072

**Published:** 2021-11-14

**Authors:** Daisuke Machida

**Affiliations:** 1 Home Economics Education Course, Cooperative Faculty of Education, Gunma University, Maebashi 371-8510, Japan; machidad0619@gmail.com; Tel.: +81-27-220-7344; 2 Faculty of Agriculture, Takasaki University of Health and Welfare, Takasaki 370-0033, Japan

**Keywords:** fruit and vegetable intake, nonmarket food, food environment, health promotion, random intercept model, cross-sectional study, Japan

## Abstract

This study investigated the relationship between prefecture-level yield of not-for-sale fruits and vegetables and individual-level fruit and vegetable intake in Japan. Data were drawn from the Japanese National Health and Nutrition Survey and National Crop Survey of 2016. Random intercept models were used for the analyses. Individual-level fruit and vegetable intake was used for the dependent variable, and prefecture-level yield of not-for-sale fruits and vegetables was used for the independent variable as a fixed effect. In addition, participants’ characteristics and health-related factors at the individual level were also put into independent variables as fixed effects. The prefectures were used as random intercepts. It was found that prefecture-level yield of not-for-sale fruits and vegetables was significantly related to individual-level fruit and vegetable intake (vegetable: B = 0.390, *p* < 0.001; fruit: B = 0.268, *p* = 0.003; fruits and vegetables: B = 0.357, *p* < 0.001). These relationships were also significant in the gender-specific analysis. Thus, the yield of not-for-sale fruits and vegetables might contribute to the intake of fruits and vegetables in Japan.

## 1. Introduction

The World Health Organization (WHO) and the UN Food and Agriculture Organization (FAO) promote increased consumption of fruits and vegetables because it can reduce the risk of certain noncommunicable diseases and the mortality these diseases entail [1]. Several meta-analyses have shown inverse associations between fruit and vegetable intake and the risk of developing cardiovascular disease or cancer and total mortality [2,3,4]. Additionally, it has been suggested that there is a positive relationship between fruit and vegetable intake and mental health [5,6]. Therefore, strategies to increase fruit and vegetable intake should be developed to promote public health.

Certain environmental factors have been known to influence fruit and vegetable intake [7]. A systematic review of environmental factors associated with fruits and vegetables intake among adults revealed an association with high household income, marriage, and good local availability of fruits and vegetables [7]. Local availability of fruits and vegetables, having a vegetable garden, and homegrown produce were positively associated with fruit and vegetable intake [7].

The influence of the cultivation of fruits and vegetables with fruit and vegetable intake has been widely reported in many previous studies [8,9,10,11,12,13,14,15,16]. Urban gardens, community gardens, allotment gardens, school gardens, and home gardens have been used as areas of cultivation [8,9,10,11,12,13,14,15,16]. Although a few study results have been inconsistent, it has been found that fruit and vegetable cultivation is generally positively associated with fruit and vegetable intake [8,9,10,11,12,13,14,15,16].

In Japan, it has been found that obtaining nonmarket food both from within and outside of the household could affect dietary intake [17,18,19,20,21,22,23,24]. Studies conducted in various locations throughout the country and in Satoyama (a traditional Japanese rural setting) showed that more nonmarket food is available in Satoyama than in other parts of Japan, and if people have more food-sharing partners, a greater diversity and quantity of food could be obtained from nonmarket food sources [17,18,19,20]. In addition, a significant relationship was found between types of land use and the ability to obtain greater amounts and varieties of nonmarket food [20,21]. Several studies have focused on vegetables in particular. One study found a positive relationship between the intake of vegetables received from neighbors and total vegetable intake [22]. In addition, it has been confirmed that vegetable cultivation and vegetable reception are related to vegetable intake [23,24]. Furthermore, in areas with flourishing fruit and vegetable cultivation, it is easy to obtain fruits and vegetables through social networks, even for individuals who do not cultivate fruits and vegetables themselves [25].

Thus, it was hypothesized that residents in areas with high yields of not-for-sale fruits and vegetables would have high intakes of fruits and vegetables. The mechanism would run as follows: the harvested not-for-sale fruits and vegetables are first consumed in the grower’s home. Then, surplus fruits and vegetables are provided to their neighbors. In addition, some neighbors who have received more than they can use may provide their excess to their other neighbors. In an ecosystem of this type, residents in areas with high yields of not-for-sale fruits and vegetables are likely to enjoy high intakes of fruits and vegetables.

This study investigated the relationship between prefecture-level yield of not-for-sale fruits and vegetables and individual-level fruit and vegetable intake in Japan. The yields of the fruits and vegetables used in this study were taken to be the amount recorded in the Japanese national statistics. This figure does not include fruits and vegetables produced in a home garden or a community garden. However, previous studies in Japan have found that 96% of farmers grow vegetables for their own consumption and 84% give away the vegetables they grow to their neighbors [24]. In addition, prefectural-level ecological studies have already confirmed a positive correlation between farmers’ yield of not-for-sale vegetables and vegetable intake [26]; however, ecological study has not clarified whether prefecture-level yield is associated with individual-level intake or not.

## 2. Materials and Methods

### 2.1. Study Design and Survey Outline

This was a cross-sectional study that used data from two Japanese national surveys.

One of these was the National Health and Nutrition Survey (NHNS) 2016 [27]. That survey investigated Japanese citizens’ physical condition, nutrient intake, and lifestyle and obtained basic data to comprehensively promote people’s health, based on the health promotion law [27]. For NHNS 2016, the subjects of the survey lived in 475 census districts, including 10 census districts per prefecture that were randomly selected (15 districts in Tokyo, due to its large population) [27]. From among these districts, Kumamoto and a part of Tottori (in total 13 districts) could not be investigated due to the effects of natural disasters and were excluded [27]. Those who lived in the subject areas and were over 1 year old were surveyed from October to November 2016 [27]. There was a total of 24,187 subject households, of which 10,745 (26,354 people) could be surveyed [27]. The NHNS 2016 was the most recent large-scale survey that examined differences in prefectures. Relative to the standard survey year, the number of subjects in that study was large, and it was considered appropriate to use its data for the examination by prefecture conducted in this study.

The other major source of data was the National Crop Survey (NCS) 2016 [28,29]. This survey clarified the actual conditions of crop production and shipment and prepared materials to formulate production targets and promote various measures in the food, agriculture, and rural basic plans [28,29]. For this study, the fruit and vegetable data were extracted. NCS surveyed the crop acreage, yield, and shipment amount of all agricultural product shipping organizations in the target prefectures [28,29]. In addition, additional surveys were conducted on the entities with crops that were not shipped through the above organizations [28,29]. The NCS was conducted every 3 years for vegetables and every 6 years for fruits in all prefectures [28,29]. In other years, the top prefectures, which accounted for approximately 80% of the cultivated area, were targeted for each surveyed item [28,29]. In 2016, the vegetable survey was conducted for all prefectures [28], but data were not collected for fruits in 2016 for all prefectures [29], so for the prefectures where the fruit survey was not conducted, data from the latest survey of all prefectures (2014) were used.

### 2.2. Variables

#### 2.2.1. Fruit and Vegetable Intake

This study used individual-level data on fruit and vegetable intake of NHNS as the dependent variables. Fruit and vegetable intake was provided and used for analyses. In addition, the total fruit and vegetable intake was calculated and used for the analyses. The unit used for all analyses was daily intakes (g) per participant.

The survey method for food and nutrition intake in NHNS was as follows: food and nutrition intake was determined by the dietary record, using a weighted method [27]. In addition, the standard amount was used for items that were used in small amounts and are difficult to weigh [27]. The survey day was set to a day of the week other than Sundays and public holidays, avoiding days when there was a special change in food intake [27]. The investigators of NHNS, who were registered dietitians, visited all of the surveyed households directly, checked the contents of the surveys, and corrected any deficiencies [27].

#### 2.2.2. Yield of Not-for-Sale Fruits and Vegetables

This study used prefecture-level yield of not-for-sale fruits and vegetables of the NCS as independent variables. The yield of not-for-sale fruits and vegetables for each prefecture was calculated by subtracting the shipment amount from the yield using data for the yield and shipment amount for each prefecture from the NCS. The shipment and yield of the NCS were aggregated by annual tonnage for each prefecture [28,29]. Therefore, in this study, dividing by the participant’s number for each prefecture [30] and the number of days per year (365) and multiplying by 10^6^, it was converted to the grams per person per day for the analysis.

NCS 2016 (vegetables and fruits) provides statistics on 55 major items (vegetables, 41; fruits, 14) produced in Japan [28,29]. However, the classification of vegetables and fruits in NCS is different from the classification used in NHNS. Therefore, in this study, the NCS data were rearranged according to the NHNS classification, following the procedure below. First, three items (potato, taro, and yam) that are not counted as fruits or vegetables in the NHNS were excluded. Then, the three items (strawberry, melon, and watermelon) counted among the vegetables in the NCS were converted to fruits, following the NHNS classification. In addition, Asian plum was classified as fruit in the NCS; however, it was counted as both of a fruit and a vegetable in NHNS. When eaten as a traditional pickle, it was classified as pickle, and it was a fruit otherwise in the NHNS. In this study, Asian plum was not included in either vegetable or fruit yield. However, the total vegetable and fruit yield included Asian plum. Finally, the total yields of 35 vegetables (cabbage, cucumber, Japanese radish, tomato, eggplant, carrot, leek, Chinese cabbage, green pepper, lettuce, onion, spinach, asparagus, green soybeans, turnip, pumpkin, cauliflower, burdock, komatsuna, sayaingen, sayaendou, crown daisy, ginger, sweet corn, celery, broad beans, bok choy, nira, garlic, Japanese butterbur, broccoli, mizuna, mitsuba, lotus root, and green peas), 16 fruits (strawberry, melon, watermelon, mandarin, apple, Japanese pear, pear, persimmon, loquat, peach, plum, yellow peach, grape, chestnut, pineapple, and kiwifruit), and 52 fruits and vegetables (all fruits and vegetables listed above and Asian plum) were used for the analyses.

#### 2.2.3. Other Items

Prefecture, gender, age, lifestyle, drinking habits, smoking habits, body mass index (BMI), and energy intake were used for the analyses. These data were individual-level data provided by NHNS [27]. Gender, age, drinking habits, and smoking habits were determined with a self-administered questionnaire [27]. Prefecture and living style were ascertained from the national census before the survey [27]. BMI was calculated from measured height and weight data. For this, the body weight (kg) was divided by the square of the height (m) to obtain the BMI (kg/m^2^). Energy intake was calculated from a survey of food and nutrition intake already written [27]. These variables were used as categories in the analyses.

### 2.3. Analyses

From the data on 26,354 individuals obtained from the National Health and Nutrition Survey, excluding those from participants aged younger than 20 and more than 79, those pregnant and lactating, and those with missing data in the items used for analysis, data from 15,046 participants aged 20–79 years (men, 6800; women, 8246) were used in this analyses. Table 1 shows the total number of participants and the number of participants by gender for each of the 46 prefectures (there are 47 prefectures in Japan; however, one was excluded because the survey was not conducted there due to a natural disaster [27]).

First, in the preliminary analyses, the yields of not-for-sale fruits and vegetables and the intake of fruits and vegetables were calculated for each prefecture, and Spearman’s correlation coefficient between these variables was calculated.

Then, random intercept models were used for the main analyses [31]. The prefectures were used as random intercepts. Fruit intake, vegetable intake, and fruit and vegetable intake were the dependent variables. These were used as interval scales (g). The yield of not-for-sale fruits, vegetables, and fruits and vegetables were used for independent variables as fixed effects. These were used as both of interval scale (g) and category (quartile categories), respectively. The trends using categorical order were also tested. Individual-level gender, age, living style, drinking habits, smoking habits, BMI, and energy intake were adjusted as a fixed effect for the analyses. The number of participants by category for each variable used in the analyses was shown in Table 2. For the model construction, first, null models wherein nothing was input for the independent variable was created, and it was confirmed whether the variance of prefectures was significant. Then, individual-level variables were placed into the models. Finally, the prefecture-level yield of not-for-sale fruits and vegetables were input into the models. The goodness of fit for each model was determined from Akaike’s information criterion (AIC) of each model [32]. For AIC, smaller values indicate better models [32].

All analyses were conducted using IBM SPSS Statistics for Windows, version 23.0; IBM Japan, Ltd.: Tokyo, Japan. Statistical significance was set at *p* < 0.05.

### 2.4. Ethical Approval

The NHNS data were obtained with permission from the Ministry of Health, Labor and Welfare. The NCS data were obtained from the Japanese official statistics portal site e-stat. The data did not contain any personally identifiable information. This study was approved by Takasaki University of Health and Welfare Research Ethics Committee (No.: 1907; approval date: 31 May 2019).

## 3. Results

### 3.1. Preliminary Analyses

In Table 3, yields of not-for-sale fruits and vegetables and fruit and vegetable intake are shown according to prefecture. For the yield of not-for-sale amounts, vegetables ranged from 1.4 to 117.0 g, fruit ranged from 0.1 to 97.2 g, and fruits and vegetables ranged from 1.6 to 183.2 g. For mean intake by prefecture, vegetables ranged from 230.2 to 345.5 g, fruit ranged from 77.9 to 143.6 g, and fruits and vegetables ranged from 309.8 to 458.3 g. The Spearman’s correlation coefficients (*p*-Value) between intake and yield among prefectures were 0.365 (0.010), 0.442 (0.002), and 0.401 (0.006) for vegetables, fruits, and fruits and vegetables, respectively.

### 3.2. Main Analyses

In Table 4, the relationships between prefecture-level yield of not-for-sale fruits and vegetables and individual-level fruit and vegetable intake, tested using the random intercept models, are shown. In addition, the AIC of each model and the *p* values of null models are shown in Table A1.

For vegetables, the regression coefficient (B) was 0.390 (95% confidence interval [95% CI]: 0.183 to 0.596) when the yield of not-for-sale vegetables was used as an interval scale. Similarly, there was a significant association in the quartile categories (Q1: Ref; Q2: B = 0.254, 95% CI = −15.660 to 16.169; Q3: B = 18.662, 95% CI = 2.962 to 34.362; Q4: B = 27.003, 95% CI = 11.087 to 42.918). The *p*-value for trend of categories was less than 0.001. Similar significant associations were found when analyzed by gender. The null model’s intercepts for all participants (*p* < 0.001), men (*p* = 0.010), and women (*p* = 0.001) were significant. For all participants, men, and women, the AICs were smaller when the individual-level variables were input from null models. The AICs were even smaller in the models that input the yield at the prefecture level.

For fruits, the B was 0.268 (95% CI: 0.099 to 0.438) when the yield of not-for-sale fruit was used as an interval scale. Similarly, there was a significant association in the quartile categories (Q1: Ref; Q2: B = −4.421, 95% CI = −13.467 to 4.625; Q3: B = 5.013, 95% CI = −3.474 to 13.500; Q4: B = 8.995, 95% CI = 0.522 to 17.468). The *p*-value for trend of categories was 0.013. Similar significant associations were found for analysis by gender instead of quartile categories for women. The null model’s intercepts were significant in all participants (*p* = 0.001), men (*p* = 0.042), and women (*p* = 0.007). The AICs were smaller when the individual-level variables were input than null models for all participants, men, and women. For the models that input the yield at the prefecture level, the AICs were even smaller compared with the other models.

For fruits and vegetables, B was 0.357 (95% CI: 0.167 to 0.548) when the yield of not-for-sale fruits and vegetables was used as an interval scale. Similarly, there was a significant association in the quartile categories (Q1: Ref; Q2: B = 8.932, 95% CI = −12.960 to 30.824; Q3: B = 21.077, 95% CI = −1.517 to 43.671; Q4: B = 32.973, 95% CI = 11.870 to 54.076). The *p*-value for the trend of categories was 0.001. Similar significant associations were found when analyzed by gender. The intercepts of null models of all participants (*p* < 0.001), men (*p* = 0.005), and women (*p* = 0.001) were significant. For all participants, men, and women, the AICs decreased in the order of null models, individual-level models, and prefecture-level models. 

## 4. Discussion

This study investigated the relationship between prefecture-level yield of not-for-sale fruits and vegetables and individual-level fruit and vegetable intake in Japan. As a result, the positive relationship between prefecture-level yield of not-for-sale fruits and vegetables and individual-level fruit and vegetable intake was found. To date, no studies have demonstrated such an association. This is the first study to identify the yield of not-for-sale fruits and vegetables as an environmental factor related to the intake of fruits and vegetables, and it may contribute to the development of a health-promoting food environment.

In preliminary analyses, it was found that there is a prefectural-level positive correlation between the yield of not-for-sale fruits and vegetables and fruit and vegetable intake in Japan. A previous study has reported a correlation of this type for vegetables, but it did not examine fruits or the total amount of fruits and vegetables [26]. This study found a prefectural-level positive correlation between the not-for-sale yield and intake for fruit and fruit and vegetables.

We found a positive relationship between prefecture-level yield of not-for-sale fruits and vegetables and individual-level fruit and vegetable intake. Many positive relationships between fruit and vegetable cultivation/receiving and fruit and vegetable intake have been reported [8,9,10,11,12,13,14,15,16,22,23,24]. In areas where there are many not-for-sale crops, much home consumption can be presumed, as well as obtaining fruits and vegetables from one’s neighbors, and, as a result, the intake of fruits and vegetables is high. According to the results of this study, fruit and vegetable intake is about 33 g/day higher in prefectures with the highest yield (Q4) than in prefectures with the lowest yield (Q1) of not-for-sale fruits and vegetables. WHO and the FAO recommend a fruit and vegetable intake of 400 g/day or higher [1]. In addition, recent meta-analyses have reported that increasing fruit and vegetable intake to 800 g/day may reduce the risk of certain diseases and mortality [2]. In Japan, nutritionists recommend 350 g/day for vegetable intake and 100 g/day for fruit intake [33]. The contribution here of not-for-sale fruit and vegetable yields may not be significant. However, even if it is a small contribution, it may raise the base of fruit and vegetable intake.

Food access is positively associated with the consumption of fresh foods, such as vegetables and fruits [7]. However, in rural areas of Japan, food access has deteriorated, due to the disappearance of local grocery stores, which are thought to have a negative impact on the intake of fresh foods, such as vegetables and fruits [34]. Even under these circumstances, it is possible that the negative impacts were mitigated by cultivating not-for-sale crops and distributing them within the region. In fact, although these data regard one island in Japan, nonmarket foods account for approximately 25% of the production price basis and around 17% of the caloric basis of food consumption as a whole [19]. In addition, approximately half of all food consumed is from nonmarket sources between the spring and autumn seasons [20]. In other words, the distribution of nonmarket foods may contribute to the healthy eating habits of Japanese people living in certain areas, where maintaining such an ecosystem may have important implications for the promotion of the health of residents. This necessitates maintaining agricultural cultivation in such areas. However, the number of farmers and cultivated land areas are declining in Japan [35,36]. The policies to revive agriculture may sustain the distribution of nonmarket foods. Furthermore, the higher the social cohesion, the higher the frequency of receiving fruits and vegetables from relatives or neighbors [25]. Therefore, programs to deepen ties among local residents may support the health of local residents through not-for-sale fruits and vegetables.

### Limitation

This study had several limitations. First, since it was conducted in Japan, its results may only pertain to that country and not to other countries or regions. In Japan, it is common to share one’s produce surplus to neighbors, so the results of this study were obtained. Therefore, results of similar studies will be different in countries and regions where there is no accepted culture of passing on one’s surplus to others. In this study, only the yield of not-for-sale fruits and vegetables produced by farmers were used as independent variables. It should be noted that the crops produced by nonfarmers in home gardens and community gardens were not included. This study also examined data at the prefecture level. However, the actual transfer of surplus often appears within smaller regional units. Finally, this was a cross-sectional study. Therefore, the causal relationship of the association confirmed in this study cannot be confirmed.

## 5. Conclusions

This study revealed the positive relationship between the prefectural-level yield of not-for-sale fruits and vegetables and individual-level fruit and vegetable intake. An environment where not-for-sale fruits and vegetables could be harvested abundantly might be contributing to the resident’s intake of abundant fruits and vegetables in Japan.

## Figures and Tables

**Table 1:** Number of participants by prefecture.

	All Participants		Men		Women	
Prefecture	*n*	%	*n*	%	*n*	%
Hokkaido	196	1.3	90	1.3	106	1.3
Aomori	376	2.5	171	2.5	205	2.5
Iwate	279	1.9	123	1.8	156	1.9
Miyagi	246	1.6	117	1.7	129	1.6
Akita	293	1.9	127	1.9	166	2.0
Yamagata	400	2.7	185	2.7	215	2.6
Fukushima	280	1.9	127	1.9	153	1.9
Ibaraki	196	1.3	87	1.3	109	1.3
Tochigi	651	4.3	321	4.7	330	4.0
Gunma	378	2.5	182	2.7	196	2.4
Saitama	460	3.1	226	3.3	234	2.8
Chiba	457	3.0	213	3.1	244	3.0
Tokyo	224	1.5	105	1.5	119	1.4
Kanagawa	199	1.3	95	1.4	104	1.3
Niigata	445	3.0	206	3.0	239	2.9
Toyama	293	1.9	119	1.8	174	2.1
Ishikawa	349	2.3	155	2.3	194	2.4
Fukui	330	2.2	145	2.1	185	2.2
Yamanashi	339	2.3	149	2.2	190	2.3
Nagano	349	2.3	147	2.2	202	2.4
Gifu	626	4.2	307	4.5	319	3.9
Shizuoka	394	2.6	179	2.6	215	2.6
Aichi	266	1.8	116	1.7	150	1.8
Mie	333	2.2	156	2.3	177	2.1
Shiga	213	1.4	96	1.4	117	1.4
Kyoto	195	1.3	87	1.3	108	1.3
Osaka	253	1.7	101	1.5	152	1.8
Hyogo	431	2.9	189	2.8	242	2.9
Nara	373	2.5	172	2.5	201	2.4
Wakayama	216	1.4	95	1.4	121	1.5
Tottori	251	1.7	106	1.6	145	1.8
Shimane	439	2.9	183	2.7	256	3.1
Okayama	313	2.1	145	2.1	168	2.0
Hiroshima	265	1.8	114	1.7	151	1.8
Yamaguchi	279	1.9	123	1.8	156	1.9
Tokushima	406	2.7	192	2.8	214	2.6
Kagawa	446	3.0	205	3.0	241	2.9
Ehime	445	3.0	195	2.9	250	3.0
Kochi	152	1.0	59	0.9	93	1.1
Fukuoka	209	1.4	93	1.4	116	1.4
Saga	306	2.0	131	1.9	175	2.1
Nagasaki	247	1.6	104	1.5	143	1.7
Oita	366	2.4	171	2.5	195	2.4
Miyazaki	366	2.4	162	2.4	204	2.5
Kagoshima	204	1.4	85	1.3	119	1.4
Okinawa	312	2.1	144	2.1	168	2.0
*N*	15,046	100.0	6800	100.0	8246	100.0

**Table 2:** Distribution of responses.

	*n*	%
Age		
20–39	2699	17.9
40–59	4986	33.1
60–79	7361	48.9
Family structure		
Living alone	1780	11.8
Living together	13,266	88.2
Body mass index		
Less than 18.5	1152	7.7
18.5–less than 25.0	9934	66.0
25.0 or more	3960	26.3
Energy intake		
Q1 (low)	3761	25.0
Q2	3762	25.0
Q3	3761	25.0
Q4 (high)	3762	25.0
Smoking status		
Nonsmoking	12,406	82.5
Smoking	2640	17.5
Drinking status		
Every day	2754	18.3
1–6 days/week	3028	20.1
3 days or less/month, rarely	3772	25.1
Never	5492	36.5
Yield of not-for-sale vegetables		
Q1 (low)	3532	23.5
Q2	3892	25.9
Q3	3809	25.3
Q4 (high)	3813	25.3
Yield of not-for-sale fruits		
Q1 (low)	3725	24.8
Q2	3502	23.3
Q3	3924	26.1
Q4 (high)	3895	25.9
Yield of not-for-sale fruits and vegetables		
Q1 (low)	3529	23.5
Q2	3937	26.2
Q3	3411	22.7
Q4 (high)	4169	27.7
*N*	15,046	100.0

**Table 3:** Yield of not-for-sale fruits and vegetables and fruit and vegetable intake according to prefecture.

	NSV	NSF	NSFV	Vegetable Intake (g)		Fruit Intake (g)		FV Intake (g)	
Prefecture	g	g	g	Mean	SD	Mean	SD	Mean	SD
Hokkaido	56.7	2.3	59.0	282.3	166.4	93.2	115.3	375.4	216.6
Aomori	85.5	97.2	183.2	312.4	180.9	118.9	148.2	431.3	256.6
Iwate	64.5	15.9	80.7	306.6	176.3	138.6	158.5	445.3	271.3
Miyagi	38.7	3.1	42.6	323.5	209.1	103.9	139.9	427.4	256.4
Akita	87.4	17.1	105.0	280.9	194.2	101.4	133.8	382.3	255.5
Yamagata	80.9	49.9	131.6	278.9	160.9	125.5	151.6	404.4	239.6
Fukushima	75.5	13.4	89.8	314.8	187.5	104.2	142.0	419.0	237.3
Ibaraki	86.2	8.4	95.1	295.3	188.0	118.7	151.1	413.9	287.6
Tochigi	54.5	6.6	61.6	278.5	182.5	81.7	112.5	360.2	231.0
Gunma	92.8	6.9	100.7	273.3	165.7	103.8	147.1	377.2	239.8
Saitama	24.2	0.8	25.1	298.5	168.0	99.0	121.3	397.6	229.4
Chiba	36.8	2.8	39.8	281.9	178.4	106.3	119.5	388.2	233.6
Tokyo	1.5	0.1	1.6	290.5	182.2	108.7	138.6	399.1	247.2
Kanagawa	5.6	1.7	7.4	292.2	156.8	92.8	123.1	385.0	220.8
Niigata	66.8	7.3	74.3	295.2	184.9	106.6	126.2	401.8	254.2
Toyama	27.4	4.1	31.6	294.6	179.7	121.5	127.9	416.0	245.7
Ishikawa	29.1	7.7	37.2	296.3	162.7	112.5	128.1	408.9	233.8
Fukui	30.4	4.0	34.8	285.5	151.3	103.6	137.1	389.0	219.3
Yamanashi	42.1	27.4	70.5	316.1	170.3	107.0	145.0	423.2	245.7
Nagano	117.0	34.3	152.0	345.5	210.9	111.8	137.6	457.3	267.5
Gifu	34.7	5.0	40.0	277.1	167.7	86.8	111.0	364.0	218.5
Shizuoka	16.7	14.6	31.6	259.5	142.9	108.0	124.1	367.5	204.8
Aichi	14.8	4.2	19.1	231.9	136.9	77.9	103.6	309.8	185.1
Mie	29.7	8.6	39.1	256.4	154.7	100.9	135.0	357.3	224.3
Shiga	28.2	3.0	31.5	260.4	158.8	105.2	132.0	365.6	233.3
Kyoto	15.7	2.1	17.8	274.5	202.6	104.9	130.3	379.4	282.3
Osaka	1.4	0.8	2.1	230.2	150.4	83.5	105.3	313.7	197.7
Hyogo	22.7	2.6	25.5	288.4	208.1	114.2	135.6	402.6	289.1
Nara	20.5	8.8	29.4	276.4	153.4	100.6	121.3	377.0	214.7
Wakayama	23.9	62.0	91.9	263.9	192.4	143.6	163.2	407.5	275.5
Tottori	86.5	23.2	110.0	287.3	148.6	117.2	120.7	404.5	204.3
Shimane	69.2	4.7	74.4	301.9	175.9	100.4	117.2	402.3	222.4
Okayama	28.9	7.5	36.8	262.4	148.3	82.3	115.1	344.7	203.6
Hiroshima	25.5	8.1	34.1	290.9	154.1	116.0	132.2	407.0	223.6
Yamaguchi	32.3	6.8	39.7	271.5	184.9	108.7	141.0	380.2	254.6
Tokushima	70.3	12.6	83.2	314.3	191.0	116.9	146.0	431.2	262.0
Kagawa	30.9	7.0	38.0	273.0	159.7	110.9	119.4	383.9	218.6
Ehime	34.0	30.7	65.0	272.3	171.3	104.2	134.1	376.5	239.8
Kochi	51.3	7.5	59.1	317.2	183.0	141.1	160.6	458.3	278.7
Fukuoka	10.2	3.2	13.6	302.3	171.0	123.1	137.0	425.4	237.2
Saga	66.5	18.5	85.5	268.2	170.6	99.8	122.7	368.0	239.4
Nagasaki	43.6	15.3	59.1	257.9	152.5	106.7	115.9	364.6	213.4
Oita	42.0	13.4	56.2	295.4	173.2	112.6	126.1	408.0	243.1
Miyazaki	60.1	6.3	67.0	294.7	190.1	109.8	131.5	404.5	257.8
Kagoshima	60.5	5.4	66.4	279.9	163.7	89.6	113.5	369.5	200.7
Okinawa	11.1	1.0	12.0	273.5	181.3	81.6	111.3	355.1	226.4
Total	45.4	13.2	59.1	285.5	174.8	105.4	130.7	390.9	239.6
Spearman’s correlation coefficient between intake and yield among prefectures (*p*-Value)				0.376	(0.010)	0.442	(0.002)	0.401	(0.006)

NSV, yield of not-for-sale vegetables; NSF, yield of not-for-sale fruits; NSFV, yield of not-for-sale fruits and vegetables. FV, fruits and vegetables; SD, standard deviation; g, g/day/participant.

**Table 4:** Relationship between prefecture-level yield of not-for-sale fruits and vegetables and individual-level fruit and vegetable intake.

	Vegetable Intake			Fruit Intake			Fruit and Vegetable Intake		
Prefecture-Level Yield of Not-for-Sale Crops	B	95% CI	*p*	B	95% CI	*p*	B	95% CI	*p*
(All participants)									
Interval scale (g)	0.390	0.183–0.596	<0.001	0.268	0.099–0.438	0.003	0.357	0.167–0.548	<0.001
Category (Reference: Q1)									
Q2	0.254	−15.660–16.169	0.974	−4.421	−13.467–4.625	0.329	8.932	−12.960–30.824	0.414
Q3	18.662	2.962–34.362	0.021	5.013	−3.474–13.500	0.240	21.077	−1.517–43.671	0.067
Q4	27.003	11.087–42.918	0.001	8.995	0.522–17.468	0.038	32.973	11.870–54.076	0.003
		*p* for trend < 0.001			*p* for trend = 0.013			*p* for trend = 0.001	
(Men)									
Interval scale (g)	0.380	0.173–0.586	0.001	0.235	0.050–0.420	0.014	0.362	0.167–0.556	0.001
Category (Reference: Q1)									
Q2	−2.554	−18.424–13.316	0.746	−2.785	−12.343–6.773	0.556	10.180	−12.376–32.736	0.366
Q3	15.946	0.128–31.764	0.048	5.314	−3.793–14.420	0.245	17.676	−5.763–41.114	0.135
Q4	23.933	8.046–39.820	0.004	10.958	1.801–20.114	0.020	33.521	11.595–55.447	0.004
		*p* for trend = 0.001			*p* for trend = 0.008			*p* for trend = 0.002	
(Women)									
Interval scale (g)	0.409	0.176–0.642	0.001	0.306	0.118–0.493	0.002	0.372	0.170–0.575	0.001
Category (Reference: Q1)									
Q2	2.104	−15.926–20.133	0.815	−5.147	−15.450–5.156	0.318	7.322	−15.876–30.520	0.527
Q3	19.828	2.020–37.636	0.030	5.049	−4.681–14.778	0.301	23.932	−0.020–47.885	0.050
Q4	29.793	11.764–47.822	0.002	7.901	−1.787–17.590	0.107	33.833	11.445–56.221	0.004
		*p* for trend < 0.001			*p* for trend = 0.039			*p* for trend = 0.001	

Random intercept model (random intercept: prefecture). Dependent variables: vegetable intake, fruit intake, and fruit and vegetable intake. Independent variables: prefecture-level yield of not-for-sale vegetables, prefecture-level yield of not-for-sale fruits, prefecture-level yield of not-for-sale fruits and vegetables. B: unstandardized regression coefficient, CI: confidence interval. Adjusted for gender (only all participants), age, family structure, body mass index, energy intake, smoking status, and drinking status as fixed effect. *N* = 15,046 (men: *n* = 6800, women: *n* = 8246).

## Data Availability

The raw data are not publicly available due to restrictions ethical.

## Appendix A

**Table A1:** Goodness of fit for each model of Table 3.

	All	Men	Women
Vegetable			
(*p*-Value of intercept of null models)	(<0.001)	(0.010)	(0.001)
Null models	197,971	90,195	107,709
Models put into individual-level variables	195,877	89,272	106,400
Models put into prefectural-level yield (g)	195,868	89,263	106,392
Models put into prefectural-level yield (trend of categories)	195,860	89,257	106,384
Models put into prefectural-level yield (quartile categories)	195,845	89,241	106,369
Fruit			
(*p*-Value of intercept of null models)	(0.001)	(0.042)	(0.007)
Null models	189,273	85,559	103,627
Models put into individual-level variables	186,557	84,501	101,950
Models put into prefectural-level yield (g)	186,551	84,498	101,943
Models put into prefectural-level yield (trend of categories)	186,548	84,492	101,943
Models put into prefectural-level yield (quartile categories)	186,534	84,478	101,928
Fruits and vegetables			
(*p*-Value of intercept of null models)	(<0.001)	(0.005)	(0.001)
Null models	207,462	94,282	113,175
Models put into individual-level variables	203,778	92,811	110,753
Models put into prefectural-level yield(g)	203,768	92,801	110,743
Models put into prefectural-level yield (trend of categories)	203,763	92,797	110,738
Models put into prefectural-level yield (quartile categories)	203,748	92,782	110,723
Series of digits: Akaike’s information criterion (*p*-Value)			
